# A Systematic Review of the Ornamental Fish Trade with Emphasis on Coral Reef Fishes—An Impossible Task

**DOI:** 10.3390/ani10112014

**Published:** 2020-11-01

**Authors:** Monica V. Biondo, Rainer P. Burki

**Affiliations:** 1 Fondation Franz Weber 1, 3011 Bern, Switzerland; 2 asdfg IT 2, 3204 Rosshäusern, Switzerland; rainer.burki@asdfg.it

**Keywords:** marine ornamental fish trade, coral reef fishes, freshwater ornamental fishes, international trade, trade monitoring, Trade Control and Expert System (TRACES), Convention on International Trade of Endangered Species (CITES)

## Abstract

**Simple Summary:**

The trade in marine ornamental fishes appears to have commenced around the 1930s, and the number and diversity of species traded remain unclear. Figures currently available are based on estimates or are inferred using limited information from various formal and trade organisations. Almost all marine ornamental fishes are wild-caught from coral reefs, and mortality rates throughout the supply chain can be high. The consequences of removing these fishes from their ecosystems are poorly understood. This article collates and examines available information including scientific studies and publications from the United Nations’ Food and Agriculture Organisation, as well as from other formal and trade organisations, in order to create a more accurate picture of the extent of commercial activities. We demonstrate that it is an almost impossible task to analyse the trade in marine ornamental fishes due to a lack of data on commerce, as well as the fact that available data for marine species is frequently combined with that for freshwater species. This review highlights the urgent need for a global monitoring system to gather accurate and timely information on the number and species of ornamental fishes in commerce, where specimens originated, and whether they were wild-caught or captive-bred.

**Abstract:**

The multi-billion dollar trade in ornamental fishes has rarely been reliably monitored. Almost all coral reef fishes are wild-caught, and few scientific analyses have attempted to elicit exact quantities and identify species involved. The consequences of the removal of millions of these fishes are poorly understood. This article collates and examines available information, including scientific studies and formal publications, in order to create a more accurate picture of this commerce. We demonstrate that it is almost impossible to analyse the trade in marine ornamental fishes due to a lack of data, and that available data for marine species is frequently combined with that for freshwater species. Figures range from 15 to 30 million coral reef fishes being traded annually, but could be as high as 150 million specimens. The global value of this trade was only estimated for 1976 and 1999 between USD 28–40 million. This review highlights the urgent need to introduce a specific harmonised system tariff code and for a global monitoring system, such as the Trade Control and Expert System already in use in Europe, in order to gather accurate and timely information on the number and species of marine ornamental fishes in commerce, where specimens originated, and whether they were wild-caught or captive-bred.

## 1. Introduction

The trade in marine ornamental fishes appears to have commenced in the 1930s, with Sri Lanka being one of the first countries to collect and export coral reef fishes for aquariums [1]. At the same time, the Shedd Aquarium in Chicago opened to the public. It was the first public aquarium in the United States with a permanent marine fish collection. The facility even featured a custom train for transporting marine fishes and seawater from Florida to Chicago [2]. The French zoologist Felix Dujardin appears to have invented the first marine aquarium in Europe in the early 19th century [3]. In the 1950s, this industry began to develop economic importance due to global air freight increasing trade opportunities [1]. By the 1990s, there had been a shift from fish-only aquariums to exhibiting reef sections in tanks [4,5,6]. Today, the industry is a multi-billion-dollar concern [6,7] involving over 50 exporting and importing countries [5,8,9,10,11].

However, very little is known about the number or diversity of fishes in commerce, and traded species typically are not traceable to their originating source because there has never been a proper monitoring system in place. It is therefore difficult to find proof for the industry’s testimonies that trade is sustainable [12,13].

Figures currently available are based primarily on (often historical) estimates or are inferred using limited information from various formal and trade organisations [4,10,11,14,15,16]. The data are also mostly based on the declared value of the ornamental fish trade and often do not distinguish between freshwater and marine fishes [10,11,12,17,18], sometimes including invertebrates [4,9]. Value highly depends on the current market price for species being traded, and to use such information to infer quantity would be unreliable [19]. It is also impossible to establish the source nation with certainty, because the country of export is not necessarily the country where the fishes were caught [11].

This systematic review aimed to locate and analyse available quantitative data on the trade in marine ornamental fishes. Given historical reports, there was an expectation that data will be lacking—a state of affairs that has been lamented by scientists and conservationists for decades [5,8,10,11,20,21,22,23,24]. This study further aims to document where possible the global scale of the industry by region and country, while identifying any major knowledge deficits. The purpose of this work is to highlight the need to establish a reliable species-level monitoring and traceability system, as well as to ensure future data distinguishes between wild-caught, captive-bred, and larval reared specimens [6,17]. We collated and analysed significant background information to help inform future decisions by policymakers with regards to data collection and monitoring the ornamental fish trade, as well as for the application of conservation programs.

## 2. Materials and Methods

A literature search was conducted via Web of Science and Google Scholar for the years 1975 to 2020 using the following keywords: “marine”, “saltwater”, and “ornamental”, “aquarium”, “fish”, “coral reef fish”, “trade” (combined with “traffic” (not illegal)), “commerce”, “industry”, and “fisheries”. For Web of Science, all papers were matched with Google Scholar to achieve a single data source. For Google Scholar, the first 20 pages from each category were analysed using a “private window”. Papers that only mentioned fishes, and not ornamental or aquarium fishes in the wildlife trade, without giving any quantitative data were excluded. In addition, a “snow-balling” approach was applied by systematically checking references from the included peer-reviewed articles [25]. All references, including those from the Food and Agriculture Organisation (FAO) and from non-governmental (including trade) organisations were considered for the period 1950 to April 2020 where available.

While this review focuses on marine ornamental fishes, freshwater species were also included because the two categories are frequently entangled in commerce, and because both are subject to major use involving similar monitoring and control challenges. Although during our search we identified one 17-year-old, non-peer-reviewed, publication by the United Nations Environment Programme/World Conservation Monitoring Centre (UNEP/WCMC) [4] that investigated the whole marine aquarium trade, including different taxa such as corals and other invertebrates, and two reviews that focused on the aquaculture of marine ornamental fishes [26,27], we did not identify any other reviews that focused on the specific issues of this paper.

Included publications were categorised by their quantitative data pertaining to traded fishes (number of fishes globally by country or region) and/or trade value (global, by country or region). Every useable quantitative information source represents a data point.

Figures for global export and import were collated and compared. Countries were assigned to a region to compare the country-specific data with data collated by region. The regions used were Africa, Asia, Europe, North America, Oceania, and Central and South America including the Caribbean (South America for convenience). Values for the three biggest importers (Europe, the United States, and Japan) [4] were compared to those for global export, as were global import values compared to those for export from the three largest exporters (Indonesia, the Philippines, and Sri Lanka) [4].

Within this study, the term “traded” defines the sum of fishes annually crossing borders between exporting and importing countries. This definition may wrongly assume that fishes cross only one border between two countries, which is not the case for Singapore, among others, that may re-export a high percentage of fishes originating from Malaysia and Indonesia. To keep the data consistent, we did not include the few datasets mentioning re-exports. The wholesale value represents the price paid by importers and the retail value is the price paid by customers at the aquarium store. If the value was not given in USD or was of an older date, we used a (historical) exchange rate converter (https://fxtop.com/en/historical-currency-converter.php). It is always considered that trade data is most often based on estimates rather than empirical studies, and that marine and freshwater fishes are habitually pooled together as ornamental fishes [1,4].

## 3. Results

A combined 546 publications from 1975 to 2019 were found using Web of Science and Google scholar. Of 284 peer-reviewed publications found on Web of Science, only 33 exclusively addressed the trade in marine ornamental fishes. Overall, 1397 figures regarding the ornamental fish trade (either volume, wholesale, or retail value) were found; 91 figures focused on the global export of ornamental fishes (freshwater and marine), 33 figures focused on the global import, 817 figures focused on export by country, and 374 figures focused on import by country. Of these figures, 22.9% concerned marine ornamental fishes. A total of 36 figures concerned the export and import by region, and 46 were identified in relation to export and import to and from Europe.

### 3.1. Countries

In this review, we found that 48 exporting and 38 importing countries were involved in the freshwater and marine ornamental fish trade, excluding countries trading exclusively in freshwater ornamental fishes (Table 1).

### 3.2. Number of Marine Ornamental Fishes Traded

**Figure 1:**
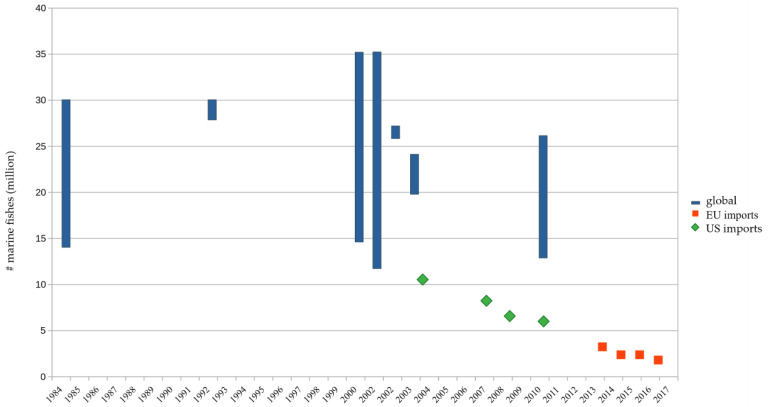
Estimated minimum and maximum number of marine ornamental fishes traded globally between 1984 and 2010 and imports to the United States for 2004/2005 (green) [5,8] and the EU (red) [11].

The referenced publications in this study estimated the global number of marine ornamental fishes traded to be between 14 million and 30 million in 1984, reaching 35 million in 2000 and 2002, then dropping to 26 million in 2010 (Figure 1). Annual imports of marine ornamental fishes to the United States for 2004/2005 represented 10.5 million in 2008, 8.2 million in 2009, and 7.3 million in 2011, and to the EU from 4.7 million in 2014 to 3.8 in both 2015 and 2016, and 3.2 million in 2017 [11] (Figure 1; see also Figure S1).

### 3.3. The Global Value

**Figure 2:**
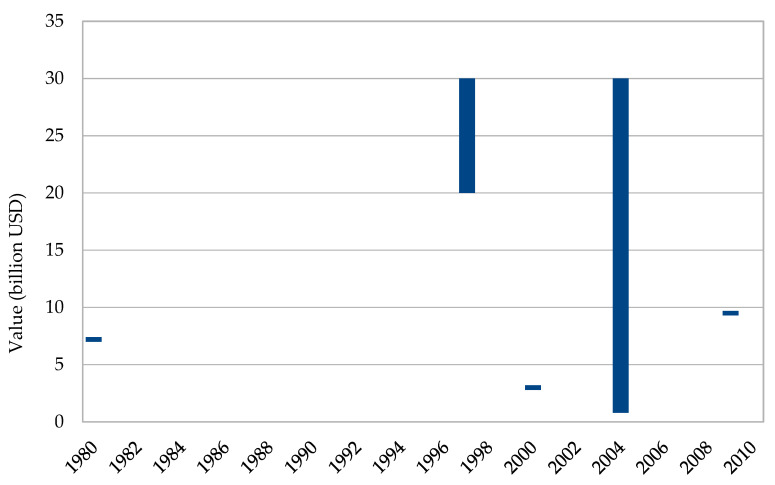
Global minimum and maximum value in billion USD of the freshwater and freshwater ornamental fish trade including accessories from 1980 to 2009 [7,16,28,29,30,31,32,33,34,35,36].

The global value of the ornamental fish trade including accessories such as tanks, food, filters, heaters, drugs, and other items for freshwater and marine fishes grew from USD 7.2 billion in 1980 to between USD 20 billion and 30 billion in 1997. In 2004, the estimates went from USD 800 million to 30 billion (Figure 2; see also Figure S2).

### 3.4. Export and Import Value

**Figure 3:**
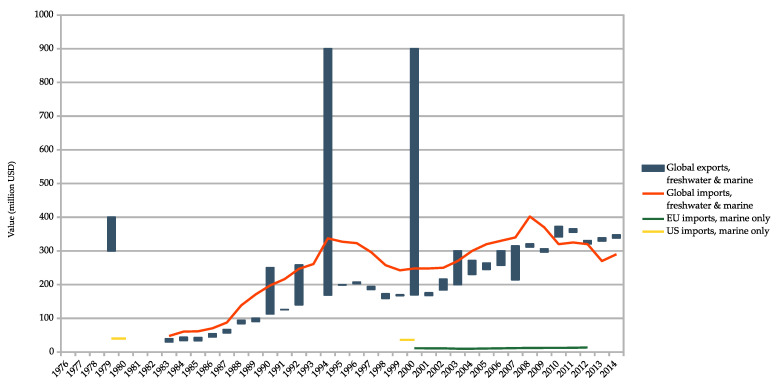
Comparison of export (minimum and maximum) and import value in million USD of the freshwater and marine ornamental fish trade, without accessories, from 1976 to 2014 [4,7,12,15,16,17,29,33,34,39,40,41,42,43,44,45,46,47,48,49,50,51,52,53,54]. For marine ornamental fishes, the global import value in 1979 and 2001 (yellow) [1,37] and for the EU between 2000–2011 (green) [38] is shown.

The export value is only available for freshwater and marine ornamental fishes and ranges from USD 21.5 million in 1976 to USD 347.5 million in 2014 (Figure 3). The wholesale import value went from USD 47.6 million in 1973 and increased steadily to USD 290 million in 2014 (Figure 3). In 1979, the global value for marine ornamental fishes was USD 40 million [1], and between USD 28–44 million in 1999 [37]. For the EU, the value was EUR 135 million (approximately USD 162 million) for the study period of 2000–2011 [38] (Figure 3; see also Figure S3).

The retail value of marine ornamental fishes was estimated at about USD 48 million in 1983 and between USD 90 million and 300 million in 2001 (Table 2).

### 3.5. Global Trade and Main Exporting and Importing Countries

**Figure 4:**
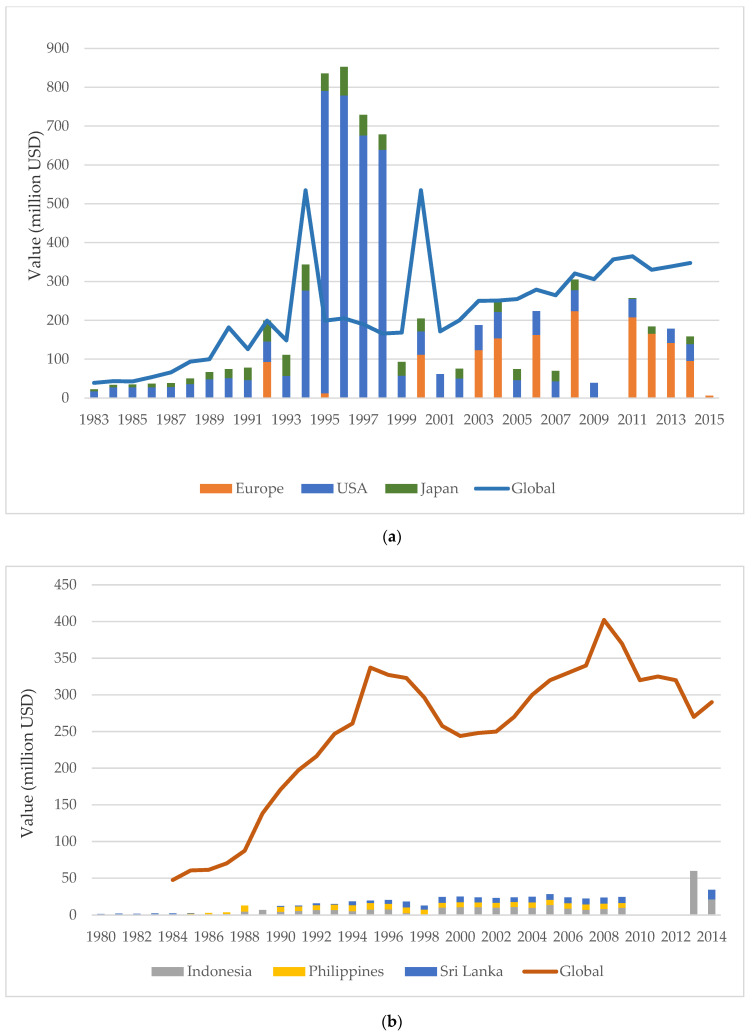
(**a**) Average wholesale value in million USD of freshwater and marine fishes for the global export compared with import to Europe, the United States, and Japan [4,7,12,15,16,17,29,33,34,36,37,40,41,43,44,46,48,49,51,52,53,54,55,56,57,58,59]. (**b**) Average wholesale value in million USD of freshwater and marine fishes for global import compared with exports from Indonesia, the Philippines, and Sri Lanka [1,12,15,16,17,20,29,33,41,44,50,52,53,60,61,62,63,64,65,66,67].

The three major importing countries of marine ornamental fishes or regions are Europe, the United States (US), and Japan, and the main exporting countries are Indonesia, the Philippines, and Sri Lanka [4]. The percentage of the global market for each respective year was determined by global exports and imports (Figure 4a). For the year 1992, as well as from 1994 to 1998, global export was similar to, or far smaller than the overall import of the three major importing countries (Figure 4a). The percentage of marine ornamental fishes from the main exporting countries compared to the global market is reflected in Figure 4b, where marine ornamental fishes represent mainly a single-digit percentage number. Indonesia exported 22.2% of ornamental fishes only once, in 2013 (Figure 4b and Figure S4a,b).

### 3.6. The Ornamental Fish Trade by Region

**Figure 5:**
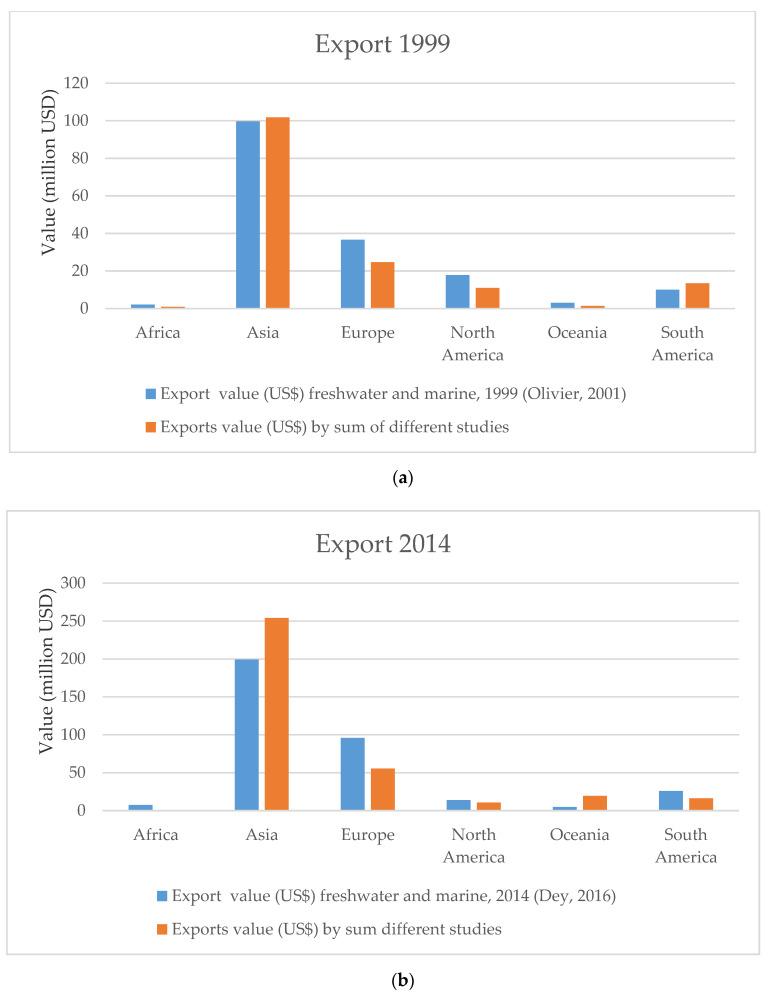

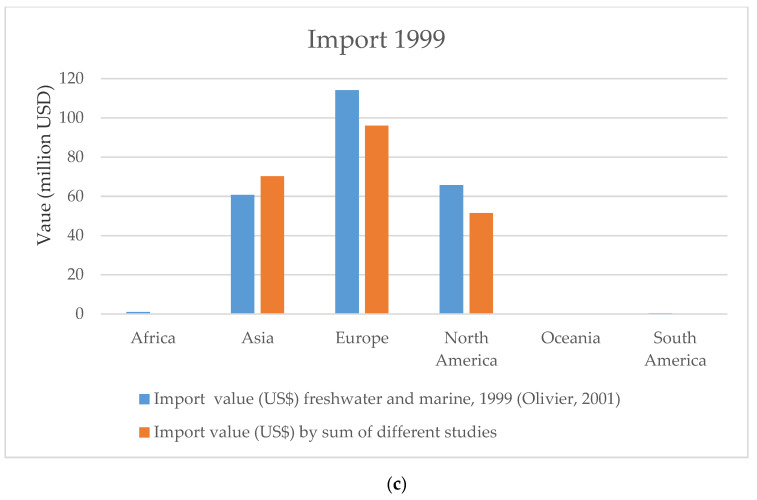
(**a**) Wholesale value in million USD of exports of freshwater and marine fishes by region in 1999 [17] compared with the sum of country exports for the same or closest year for which data were available. (**b**) Wholesale value in million USD of exports of freshwater and marine fishes by region in 2014 [16] compared with the sum of country exports for the same or closest year for which data were available. (**c**) Wholesale value in million USD of imports of freshwater and marine fishes by region in 1999 [17] compared with the sum of country exports for the same or closest year for which data were available [12,15,16,17,18,29,42,44,68,69,70,71,72,73,74,75,76,77,78,79,80,81,82,83,84,85,86,87,88,89,90].

Data for freshwater and marine fishes by region from Olivier [17] and Dey [16] was compared with data available from other countries (Figure 5a–c). For some regions, such as Africa, few figures were available. For 2014, data for region and country often stem from the same publication [16] (Figure 5c and Figure S5a–c).

## 4. Discussion

More than half of the vertebrates in the wildlife trade are fish species [91]. In the United States alone, 160 million ornamental fishes are kept in aquaria, of which almost 20 million are of marine origin [92]. The United States imports about 10 million marine specimens annually [5,8], which indicates an average life span of two years per fish. The ornamental fish industry strives to be perceived as sustainable [13], but there are only a few case studies about sustainable fisheries, and sustainability is mainly understood in terms of the livelihoods of local fishermen and villagers [13].

One of the underlying problems concerning trade evaluation and sustainability is that limited data are available regarding the commerce in marine ornamental fishes, including their diversity and place of origin. Where figures are available, they must be treated with great caution because they may be inferred [93] or, as is the case for FAO data for example, emerge from only a few traders who are voluntarily willing to provide information [4,12].

The lack of trade information such as number and diversity of specimens traded, the methods of collection, or their exact source has been lamented for over 30 years [1,5,8,10,11,18,37,41,62,94,95] because trade is likely to have an impact on affected marine environments [12]. For the marine ornamental fish trade, the problem is amplified due to the volume and diversity of species being caught in the wild [4,5,10,11,12,18], as well as the lack of information on their exact source and location of captures [5,8,10,11,18]. There are many stakeholders involved, including fishermen, intermediaries, exporters, and importers, which makes the traceability of this trade challenging [96]. Another concern is the mortality of specimens within the supply chain [6,7,12,37,97,98,99,100]. Losses incurred throughout the supply chain are not considered, thus masking the numbers even further.

From 1976 to 2014, only 1397 data points were found that represented figures from the monitoring categories of importing or exporting countries’, with these being the number of fishes, wholesale value, and retail value. If each country identified as a contributor declared one data point per category annually from 1997 to 2014, then 10,062 data points would have been identified (39 years × 3 datapoints × 86 countries). However, only 14% of these proposed data points were identified, with a mere 3% of the total being marine-specific, which is further compounded by the lack of data beyond 2014.

This review found that between 13 million and 35 million marine ornamental fishes may be exported annually (Figure 1). However, because the overall trade in ornamental fishes is estimated to be 1.5 billion, and marine fishes are said to make up 10% of this figure [4,12]; this would indicate 150 million marine ornamental fishes are being traded annually. Far larger volumes would appear to be involved than those shown in Figure 1. Ploeg and Bassleer advise caution with their figures because they are based on voluntarily provided information [34,101,102]. Ploeg was able to demonstrate that none of the available statistics were reliable for Dutch figures for 2004 [34].

The best estimates for the number of marine ornamental fishes traded globally appear to come from the Global Marine Data Base (GMAD). This database was introduced in 2002 and averaged 26 million marine ornamental fishes traded for a single year [14]. The database collected importer data for 1998 and 1999, and exporter data for 2000 and 2001, with 41 contributing companies, and the global number of fishes was inferred by searching the total number of companies in the member logs of different trade organisations and the Internet (total of 266 companies). Unfortunately, data entry ceased after one year. The only possibility to verify GMAD data is to compare the information with Wood [1,37]. Wood estimated the number of coral reef fishes being traded to be between 28 million and 30 million for 1992 [37] by extracting figures from the relevant government export and import statistics, which would seem to be more accurate. However, the author mentions that not all figures were reported, or were partly combined with freshwater fishes, or were extracted from figures given by weight, which included the weight of water present. GMAD [93] and Wood [37] seem to agree that the number of marine ornamental fishes traded totalled around 30 million between 1992 and 2000.

The best numbers for Europe come from an evaluation of custom figures collected by the Trade Control and Expert System (TRACES), which found an average of 4 million marine ornamental fishes imported annually between 2014 to 2017 [11]. These figures must also be treated with caution. Figures from 2007 and 2011 for the United Kingdom (UK) from the Animal Health and Veterinary Laboratories Agency, which also derive from TRACES, reported that the United Kingdom imported approximately 40 million freshwater and marine ornamental fishes annually—figures that could include the aforementioned 4 million marine specimens (10% of all ornamental fishes are of marine origin). With this estimate being for the United Kingdom alone, one could assume that Europe must import many more marine ornamental fishes [103]. For the United States, approximately 10 million marine ornamental fishes are declared as being imported annually [5,8]. It is still assumed that Europe, the United States, and Japan are among the largest importing countries [4,38]. The approximately 14 million fishes imported annually by the EU and the United States fit into the annual world trade assumed above of between 13 million and 35 million marine ornamental fishes. No recent trade figures appear to be available for Japan and certain countries (e.g., China) or regions (e.g., the Middle East [102], Africa, or South America), and with their increasing commercial gravitas and number of public aquariums [104,105], they are likely to have increasing import volumes of marine ornamental fishes.

Assuming that the market share in value is the same for volume (i.e., number of fishes), this would result in a global trade of approximately 70 million marine ornamental fishes annually using the United States figures of a 14.3% market share (10 million specimens × 100/14.3) [16]. The same calculation for the EU with a market share of 35% [16] would result in approximately 11 million marine ornamental fishes being traded annually. This could mean that the global number of traded marine ornamental fishes is between 11 million and 70 million a year. A median volume of 30 million marine ornamental fishes (Figure 2) is suggested, but the range is still very wide, and this discrepancy is difficult to explain. Perhaps the discrepancy is in part explained by the fact that, according to Biondo and Rhyne’s figures [5,8,10,11,18], the European market share is approximately half that of the United States and not twice that estimated by Dey [16], and partly by the fact that Americans import many more inexpensive fishes than Europe [102].

We endeavoured to consistently interpret the available figures, although in some sources it is unclear what the stated global trade signifies. The few figures regarding the value of trade arise from different categories, for example, marine fishes only; freshwater plus marine; fishes plus accessories (e.g., aquaria and food); or recorded at export, import or retail.

The global trade has increased in value over the decades, and aquatic organisms for home and public aquariums, along with associated equipment and accessories, is today said to be a multi-billion dollar industry [7]. In the 1980s alone, the global ornamental fish industry was estimated to be worth USD 7.2 billion [28] and in 1997 it had reached between USD 20 billion and 30 billion [16,29,30,31,32]. The discrepancies grew extensively in 2004 when the total value was estimated to be between USD 800 million and 30 billion annually [7,33,34,35] (Figure 1).

Declared export value figures for ornamental fishes (freshwater and marine) without accessories more than doubled from 1999 to 2014 (Figure 3). However, these figures also vary greatly, and are questionable. For example, stated figures varied from USD 21.5 million in 1976 [12] to a rise to between USD 300 million and 400 million [50] just three years later, then to fall to less than USD 40 million in 1983 [17,39] and reaching USD 900 million in 1994 and 2000 [29,33].

Import values are almost always higher than the export values, which makes sense, although these values are not twice as high, as is stated in literature [37]. The figures for export and import from 2011 onwards may be subject to greater uncertainty because the import figures are lower than the export figures. We are of the opinion that the wide range for the year 1979, with values between USD 300 million and 400 million [50], as well as the value of USD 900 million in the years 1994 and 2000 [29,33], have to be disregarded as they do not seem plausible.

Global import values of freshwater and marine ornamental fishes averaged USD 280 million annually between 2000 and 2014, with a European market share of approximately 60% [16], which resulted in USD 168 million for Europe. We endeavoured to validate this value using Leal [38], who found that the EU imported marine ornamental fishes from 2000 to 2011 for about USD 13.5 million annually. If marine ornamental fishes represent 15% or greater [16] of the trade value, then this would result in at least USD 90 million of freshwater and marine ornamental fish imports to the EU (13.5 × 100/15). These data would suggest a global import market of freshwater and marine fishes of about USD 150 million (the EU has a market share of 60%). This figure seems to us to be plausible because the relevant data are in a similar order of magnitude.

In addition, between 2014 and 2017, the EU imported 4 million marine ornamental fishes annually [11], which would represent USD 3.5 per fish on the basis of the USD 13 million overall value stated by Leal [38].

In 1996, the value of imported fishes (without accessories) reached over USD 320 million [40], whereas in 2008, the value reached over USD 400 million [16], only to again fall to USD 290 million in 2014 [16] (Figure 3). We compared both the export and import statistics and checked for credibility, and at least the commercial activity trend was similar—although the figures do not exactly match. Large discrepancies were already found in the UNEP study [4], where export and import figures did not match.

There is a greater uncertainty regarding the value of traded marine ornamental fishes because the rare estimates of trade value are only available for freshwater and marine fishes combined. Depending on species involved, marine fishes probably hold proportionately greater value—perhaps by 15%—in the market [16]. The retail value of the trade in marine specimens (fishes and invertebrates) may be between USD 25 million and 300 million [1,37,55]. Considering that the retail value of fishes is estimated to be double or three times the wholesale value [37], the retail values seem too low in the case of USD 25 million and too high in the case of USD 300 million.

Up to 135 countries are involved in the freshwater and marine ornamental fish trade [12,15,34]. The present study identified 86 countries (48 exporting and 38 importing) as being involved in freshwater and marine ornamental fish trade. Almost 50 countries were involved in the export of marine ornamental fishes alone. This multinational involvement was present in the 1980s and continues today [1,10,11,16], and it is very probable that additional nations may be involved because the trade appears to be expanding to small island states in the Pacific [101].

Comparing between the global export data for ornamental fishes and the import data of the main recipients (Europe, United States, and Japan), one observes that fluctuations are relatively high (Figure 4a). For the year 1994 to 1998, the data originated from the U.S. Fish and Wildlife Services (USFWS) and not from customs. Importantly, USFWS data do not exclude shipments with values less than USD 1251, but customs does exclude this information. The excluded information can represent a significant share of the total number of shipments entering or leaving a given U.S. port. Annual import and export values reported by USFWS are at least one order of magnitude higher than those reported via customs, although the exact reason for this difference has not been identified. [57]. It can be argued that flawed data relating to a single large importer could significantly alter the global value as provided in, for example, Figure 4a. Studying the data, we held the impression that other authors have used U.S. customs documents to calculate import values for the U.S. market. It is confusing that data from 1999 onward is as low as in the years prior to 1994. Since 2010, the European trade value appears to be decreasing, although we have figures regarding value only until 2014. Data for the number of marine fishes from 2014 to 2017 also show a decline [11].

Comparison between the global import and export data of the main marine ornamental fish exporters Indonesia, the Philippines and Sri Lanka suggests that these nations constitute trade contributors of only a single-digit percentage. This may be due to the fact that these countries primarily export marine fishes [101] and thus represent a relatively small portion of the ornamental fish trade (Figure 4b).

A rough comparison of data by region [16,17] with data available from other countries was produced to assess whether figures from these two sources are plausible, and this appears to be the case (Figure 5a–c). Exports from Europe are high and probably involve captive-bred freshwater fishes [106]. Furthermore, there is very little information regarding China, Japan, or other Southeast Asian countries, as well as Africa and South America.

Ninety-nine percent of marine ornamental fishes are wild-caught (from coral reefs), and thus only 1% are captive-bred [27,107]. Mortality rates in the supply chain [7,12,37,97,98,99] can be very high, and the fact that fishes are often still caught illegally with the use of poison such as cyanide [6,97,108], as well as the threatened state of the coral reefs [109,110], makes it seem possible that the marine aquarium industry is putting further pressure on relevant ecosystems [45,97]. It has been shown that the aquarium fish trade can have negative impacts on coral reefs and their occupants [6,95,97,111]. The current trade could potentially represent a major concern, not least because the diversity of species in commerce appears to have increased steadily over the decades. Of the 4000 known coral reef fishes [112], only 200 species were reported as being in trade in 1991 [45], whereas between 2008 and 2011 the United States alone imported more than 2300 species, excluding species imported from U.S. waters or coral reefs in U.S. territories [8]. The United States has many endemic marine ornamental fishes, for example, over 80 species in Northern Hawaiian waters [113]. In Europe at least 1334 marine species are in trade, possibly more because customs information allows only the family and not the species name to be declared [10,11,18], and Switzerland alone imported 440 species of coral reef fishes in 2009 [18].

Furthermore, over one-third of the species imported into the EU between 2014 and 2017 [11] and almost 45% of all coral reef fish species in 2018 were not evaluated by the International Union for Conservation of Nature (IUCN) Red List as there is insufficient scientific data concerning species’ biology and ecology [18]. In the 1980s, it was thought that captive-bred marine ornamental fishes would soon be widely available [28], yet today only 34 species are readily obtainable in aquarium shops in the United States [23,28].

Some attempts have been made to increase transparency in the ornamental fish industry. One attempt was the Marine Aquarium Council (MAC) label that was established in 1998 to ensure traceability, good practice, and sustainable schemes of ecologically and socially responsible fishing, but this has been inactive since 2008 [114].

The European Statistical System (EUROSTAT) compiles the value of imported fishes by weight in kilograms (including the water in which the fishes are transported), and thus the data do not represent the number or diversity of fishes. Other data collection systems (for example FAO) are also uncertain because they are not compulsory and not species-specific.

The Trade Control and Expert System (TRACES) could potentially provide an adequate mechanism for monitoring commerce in ornamental fishes. TRACES assists with the exchange of information between veterinary authorities, ensures traceability, and contributes to rapid assessment measures in the event of disease outbreaks. TRACES is in use in 85 countries with 42,000 users, and is available in 34 languages [115]. Many of these parties already either export or import marine ornamental fishes, and, with some technical adjustments, the TRACES data collection system could be modified to collate enough data to cover the largest part of the trade [11].

TRACES would not only quantify but also qualify this trade by collecting data at the species level, and could also require specification as to whether a species was wild-caught, captive-bred, or captive-reared; thus, all fishes (and other vertebrates) would be traceable to their source. Today, although much of this information is already collected, data acquisition is non-compulsory and therefore arbitrary and highly incomplete. Furthermore, the aquarium industry has clearly stated its interest in using a tool such as TRACES to monitor commerce, as well as to develop the sustainability of this trade [49].

TRACES uses the harmonised system code (HS code) 03011900 “other fishes” for marine ornamental fishes. This code, or another unique number, should be introduced worldwide, together with the requirement to list the species-scientific name and the number of specimens [116], which would greatly improve record-keeping because customs documents from many countries are already available electronically.

Relatedly, the Convention on International Trade in Endangered Species of Fauna and Flora (CITES) is an international convention designed to ensure sustainable international trade in threatened animals and plants. However, trade is monitored only where species are listed in the relevant appendices. CITES listing can provide a significant step forward for certain species because data collection becomes compulsory, and non-detriment studies would be mandatory to ensure a sustainable trade. Moreover, should uncertainty of sustainability arise, then a “significant trade review” (CITES process to ensure that trade in animal species is not detrimental to their survival) needs to be conducted. The same principle is involved for captive-bred or captive-reared species. In 2019, the 183 member states of CITES agreed to commence in scrutinising trade in marine ornamental fishes (https://cites.org/eng/dec/valid17/82264). Regardless, government cooperation is urgently required to resolve monitoring and controlling issues [117]. We recommend that the initial step for governments is to compile data from relevant export and import statistics pertinent to all relevant nations trading in marine ornamental fishes (as per Wood’s research) [37].

## 5. Conclusions

This study highlights the fact that there is a major lack of information regarding the marine ornamental fish trade, stressing the need for a compulsory and comprehensive monitoring system that collects information regarding the number of traded specimens; diversity of species; country of origin; and source, i.e., whether specimens are wild-caught, captive-bred, or captive-reared. As a result of this review and analysis, we show that the numbers and species diversity of marine ornamental fishes in trade are only vaguely known, and speculation surrounds both their origins and commercial volumes.

It is very surprising and concerning that in the 21st century an entire group of vertebrates (in particular, 4000 known coral reef fishes) can be traded with few regulatory and monitoring systems in place. For decades, conservationists and other scientists have demanded comprehensive regulation, monitoring, and control of the ornamental fish trade, and we agree that these measures are indeed long overdue and require urgent implementation. Towards this action, we recommend that TRACES constitutes an excellent tool that, with a few modifications, as outlined previously, as well as appropriate political will, could be used almost immediately for the marine ornamental fish trades to systematically monitor and collate relevant data.

## Figures and Tables

**Table 1:** Countries and regions of export and import of ornamental fishes that included marine fishes between 1962 and 2017.

	Export	Import
**Africa**		
Egypt	x	
Eritrea	x	
Kenya	x	
Nigeria	x	
Tanzania	x	
**Asia**		
China	x	
Hong Kong	x	x
India	x	x
Indonesia	x	x
Japan	x	x
Malaysia	x	x
Maldives	x	
Philippines	x	
Singapore	x	x
Sri Lanka	x	
Taiwan	x	
Thailand	x	
Vietnam	x	
**Europe**		
Austria		x
Belgium	x	x
Bulgaria		x
Cyprus		x
Croatia		x
Czech Republic	x	x
Denmark		x
France		x
Germany		x
Greece		x
Hungary		x
Israel	x	x
Ireland		x
Italy		x
Luxembourg		x
Malta		x
Netherlands	x	x
Norway		x
Poland		x
Portugal		x
Romania		x
San Marino		x
Slovakia		x
Slovenia		x
Spain		x
Sweden		x
Switzerland		x
United Kingdom		x
**North America**		
Canada		x
USA	x	x
**Oceania**		
Australia	x	x
Cook Islands	x	
Fiji	x	
Kiribati	x	
Marshall Islands	x	
Palau	x	
Papua New Guinea	x	
Tuvalu	x	
Vanuatu	x	
**South America**		
Bahamas	x	
Barbados	x	
Belize	x	
Brazil	x	
Colombia	x	
Costa Rica	x	x
Cuba	x	
Curacao	x	
Dominican Republic	x	
Guadeloupe	x	
Haiti	x	
Honduras	x	
Martinique	x	
Peru	x	
Puerto Rico	x	
Venezuela	x	

**Table 2:** Estimated retail value (USD) for marine ornamental fishes from 1983 to 2001 [1,37,55].

Year	Retail Value in Million USD for Marine Ornamental Fishes	
	Min	Max
1983		48
1985	25	40
2001	90	300

## Supplementary Materials

The following are available online at https://www.mdpi.com/2076-2615/10/11/2014/s1, Figure S1. Estimated number of marine ornamental fishes. Figure S2. Global value. Figure S3. Comparison of export and import value. Figure S4. (a,b) Average wholesale value export and import. Figure S5. (a–c) Wholesale value by regions.
